# Servant Plague

**Published:** 1857-08

**Authors:** 


### HALL'S JOURNAL OF HEALTH.

OUR LEGITIMATE SCOPE IS ALMOST BOUNDLESS: FOR WHATEVER BEGETS PLEASURABLE AND HARMLESS FEELINGS, PROMOTES HEALTH; AND WHATEVER INDUCES DISAGREEABLE SENSATIONS, ENGENDERS DISEASE.

VOL. IV.] AUGUST, 1857. [NO. VIII.

WE aim to show how disease may be avoided, and that it is best, when sickness comes, to take no medicine without consulting an educated physician.

### SERVANT PLAGUE.

THE utterly trifling character of many of our cooks, nurses, and housemaids, is one of the chief ingredients of domestic inquietude. It makes more mad women, more grumpy men, and keeps up more incessant annoyance in a family, than all other causes combined. It worries the wife, saddens the husband, breaks in upon the enjoyment of the children, and keeps multitudes of houses in a state of irritation and unrest, which eats out half the enjoyment of domestic life. No wonder the disappointed husband flies to the club-house, the billiard table, the whist party, or the "saloon" of gilded guilt, where external gorgeousness pleases the eye, and delusive drinks steal away the senses—while the high priests of the faro-bank and the card-pack steal the purse; and lower down still, where painted beauty steals the virtue, leaving nothing to tell the tale but the bloated face, the bankrupt counter, and the blasted reputation. No wonder that young girls stray away to the ball-room, and young boys to the theatre or the negro opera. No wonder that the overtaxed wife fails, and fades, and pales away,—or, mounting a mettled nag, rows her husband up salt river!

Let then, all independent housekeepers make a strike for higher compensation for the monthly seven dollars paid the nurse or chamber-maid, and the eight dollar cook, who can eat "pastry" but can't make it, who expects you to get all your bread at the baker's, and "stipulates" that your washing is to be hired out; who gives you to understand that she must have loaf sugar in her tea and coffee, as "brown" gives her the headache; who must have green tea for breakfast; that the front gate must not be kept locked, as it looks as if you didn't want any of her friends to come and see her; whose "perquisites" are all the "soap fat," for which she gets two cents a pound, made up of fresh lard costing sixteen cents, and butter at thirty-four cents, with an occasional slice from the sixteen cent ham, and the fourteen cent fresh pork—it being "understood" that besides the weekly afternoon out, which extends from three o'clock P. M. to midnight, she is to have a chance at church on Sunday, and the occasional christenings and funerals, averaging, as every housekeeper may conjecture, once a week, on which occurrences you need not expect to see them until next day after breakfast, stupefied with want of sleep, worn out with the all-night carousal, and far more fit for a bed in a hospital than for the duties of a house servant. We have observed these impositions literally in families, with many others as onerous; happily, for our own selves, such experiences have not been ours, having had the "help" of an excellent colored family, by birth, "Jersey," by profession, Methodist. And thus *we* navigate, not burdening the reader with too many particulars, rather designing suggestive hints. All our regulations are by "*mutual consent*,"—so there is no hardship anywhere. Washing, bread-baking, pastry cooking, every thing is done in the house. The washing of nine persons is completed by Monday noon. All our bread is made of flour or meal, not of cream of tartar, soda or saleratus; such things do not enter our dwelling, except in the stomachs of the people whom the use of such things brings to our office, so if we do not live *on* saleratus we live *by* it. Saleratus is a valued friend of ours, wherever met, except in our own daily food. Our girls visit nobody, from one year's end to another; they want nobody to visit them. Until nine at night, they read our exchanges or sew for themselves. At nine they go to bed, we setting the example, summer and winter, except on necessary occasions, for we make it an inflexible rule in our own family, to be a slave to no habit or regulation, good or bad, leaving ourselves to be governed in all cases by the circumstances of the moment—this only is rational liberty. Thus, we never require a promise from any member of our family, child or servant. We endeavor in plain, and courteous, and kindly language, to let them know our wishes, and if not met, we never fail to notice it with the same kind courtesy, and to require a satisfactory reason. It is perfectly marvellous how soon children and servants fall into line, and scarce once in a month, we do not remember that once in a year, has it been necessary to repeat a wish a second time to one of our girls. Can't exactly say the same of our 3, 5, 7 and 9-year olds. Line upon line, and now and then the "tincture of birch" seem to be "indicated," as doctors write. Our girls do not expect to be waked up of mornings. They wake themselves regularly at day-light, and a radius of five minutes includes the meal calls of a year. In short, the faintest scintillation of upbraiding is not needed, nor remembered in a twelvemonth. The "*range*" is dampered down, the moment the food is placed on the table. The coal in the cellar is taken up from the cellar floor, and the dust swept after, so at the end of a whole fall, winter and spring burning, there is not a peck of coal dust in the cellar. Not an ounce of ash or cinder is ever thrown there. Not a thing in the house is kept under lock and key. Sweetmeats, pastry, tea, coffee, sugar, every thing is open. We say to them, what you want take freely. Their faithfulness merits it. It is a pleasure thus to treat them. By a kind of instinct or natural tending, we cannot remember when we have given a positive order on any subject. They are so willing to do anything requested, that we find ourselves putting it in the form of request—"Mightn't it be well to have a turkey for dinner, to-morrow?" "Would it be much trouble to have some buckwheat cakes in the morning?" "No, sir." "But have you any rising, and it is a rainy night?" "No, sir, none in the house, but I can easily go for it." Now, my poor afflicted New York housekeeping sister, don't you fairly love our girls, "unsight, unseen?" As for ourselves, we respect them, and respect their lady-like mother, and their aged clerical father, for the wisdom and tact which they have exhibited in bringing up their houseful of children, all good, not a "black sheep" among them.

Said we to wifey one day, "I see no charges for yeast, for I can't tell when, what is the reason?"

"Oh, Hannah says the proceeds of the 'soap fat' pays for that."

Not long ago, we noticed that Saturday's turkey, as left at the close of dinner, appeared on the table next day (for we aim to have no cooking on Sundays, except coffee, tea, and a little warming-up sometimes) undiminished; on inquiring the cause, the cook stated that it was smaller than common, and she and her sister thought they would eat something else, that there might be plenty for our dinner to-day. With such considerations and self-denial for the benefit of their employers, our esteem is compelled—while we are daily thankful, for the good fortune which has befallen us in respect to our "*help.*"

The Croton Aqueduct Department is a debtor *to Hannah's* consideration. She had read in the daily papers, that in consequence of the water faucets being allowed to run too freely, the supply in the reservoir was alarmingly low, and in case of any large fire, would be disastrous. This was in early February, and as soon as the weather became very mild, the water was shut off in the kitchen entirely. The consequence was, that next morning we had no water. She did not know that the cold had penetrated the ground, that it was frozen in some places to the depth of several feet, and that it would require at least a week or two of moderate weather to unthaw the ice beneath the surface. It may be of service to remark here, that it is a useless and reprehensible practice to let the water run during the night in a full stream, in order to prevent freezing. A continuous stream, which prevents the water breaking into drops for four inches below the nozzle of the faucet, is amply sufficient in our house to prevent freezing in the coldest weather. It will be worth the price of a year's subscription to any of our city subscribers to know, that our plumber, ENNEVER, 180 Third Avenue, thawed out our water-pipe in half an hour, by one of those simple, yet philosophical ideas, which the thoughtful mechanic so often throws off from the brain, without any tearing up of the pavement, digging frozen earth with crow-bars, and keeping fires burning in the holes for days and nights together. One of our neighbors was three days rectifying his hydrant, at an expense of fifteen or twenty dollars, to say nothing of the inconvenience of being without water, and having no cooking in the family for all that time, except with great danger or trouble. But we have digressed, and perhaps, transgressed, but as we wish to be practically useful in the way of preventing as many of the annoyances of life as possible, we will offer no apology, but proceed to "resume the thread of our discourse."

"Once ago," as our little Bob says, when he wishes to speak of a past occurrence, Hannah wished a vacation, and "Mary" fell to our lot for a few months, when she became ill, and an opening was made for Hannah's welcome return. Now Mary was a maiden lady from the north of Ireland, who never would confess beyond forty, although really a "quarter" short. Mary was a *nondescript.* She had no visitors, and never would visit. Worked from morning until night, if there was anything to do, and yet hated work with a perfect hatred. She would talk you to death one day, if you would listen, and for the following week utter nothing but a "yes," or "no," and not even that, unless spoken to. Never behind hand in anything. Not a spoonful of dust or dirt anywhere within her range. Punctual to a minute with her meals, the year round. Honest as could be desired, careful to save every ounce of food, never to give an atom away, as she agreed with our inflexible rule, not to give or receive, purchase or sell, to the amount of a morsel or the value of a penny at gate, or basement, or hall door. This, reader, is a kink of ours, as being neither profitable nor beneficent. For what you have to give in money, food, or cast-off clothing, let it be placed in the hands of the various societies, whose members make it their business to bestow on worthy objects, and on those alone. It is the impostor, or the unworthy idler, who begs from door to door, three times out of four.

But Mary had her drawbacks,—at least one worth speaking of. She was a perfect wretch for temper and contrariness. The children hated her—absolutely hated her. She never was willing to cook anything else than what she felt like eating herself, and the very best of everything she would keep back for her own use—the sweetbread of veal, the breast of the spring chicken, the liver of the turkey. In short, she knew what was good to eat, and would have it. Mary's temper met wifey's temper, and the amalgamation resulted in "continued fever," as doctors love to speak. Wifey talked the fastest, but Mary beat her in the sound. How fast and loud it went, how the sparks flew, how the eyes snapped, how the lips quivered, and the cheeks of the respective parties colored, we cannot pretend to describe. But, somehow or other, "Jersey" would always beat "Erin," and Mary would come up to our office in tears. "Doctor, I'm a lone woman; ye're a gentleman, so ye is, an I kid liv wid ye til I was as old as Methuzlejig, and the madam is the kindest-hearted lady iz iver I knowd, sept when she aint 'cited. Please, sir, I'ze cum to give ye warnin."

"Very well, Mary, whenever you are tired of us, your wages are ready; but mind, you go away yourself—we don't send you off."

For the next month or two, Mary would be a model. At last, sickness incapacitated her from performing her duties, and we saw she needed rest. Taking her all in all, Mary was a worthy woman; and to any lady housekeeper, who was quietly firm, and had no will of her own, she would be invaluable.

Looking over the whole subject of servants, we should consider their ignorance, and pity and bear with them; treat them courteously; keep them at a respectful distance; require system and punctuality in all things. Let them feel that your will is law, that no injunction is to be given beyond the second time, and that their whole duty to you will be exacted by you, as much as the last cent of their wages is exacted by them. Patiently instruct them, and always speak kindly, or if in reproof, even that may be mildly done; and when they leave, let certificates be given, whose literal truth shall do even-handed justice to the one who has left you, as well as to the household where she may next find employment. Such a course, would work a domestic revolution within a year.

